# Application of 3D Ultrasonography in Detection of Uterine Abnormalities

**Published:** 2012-02-20

**Authors:** Firoozeh Ahmadi, Fatemeh Zafarani, Hadieh Haghighi, Maryam Niknejadi, Ahmad Vosough Taqi Dizaj

**Affiliations:** Department of Reproductive Imaging, Royan Institute for Reproductive Biomedicine, ACECR, Tehran, Iran

**Keywords:** Müllerian Duct, Anomalies, Endometrial Diseases, 3-Dimensional, Ultrasonography

## Abstract

Structural pathologies in the uterine cavity such as müllerian duct anomalies (MDAs) and intrauterine lesions (fibroids, polyps, synechiae) may have important roles in subinfertility, implantation failure and pregnancy outcome. Various imaging modalities such as hysterosalpingography (HSG), sonography, laparoscopy and hysteroscopy are used in the evaluation of MDAs and intrauterine lesions. Recently, three-dimensional ultrasound (3DUS) has been introduced as a non-invasive, outpatient diagnostic modality. With increased spatial awareness, it is superior to other techniques used for the same purpose.

## Introduction

Detection of uterine abnormalities has been the focus of recent research in gynaecology. Structural pathologies in the uterine cavity such as müllerian duct anomalies (MDAs) and intrauterine lesions (fibroids, polyps, synechiae) may have an important role in subinfertility, implantation failure and pregnancy outcome. As a result, screening for uterine abnormalities is a part of routine clinical investigations of women who have histories of infertility, recurrent miscarriages and early preterm labor (1). Two-dimensional transvaginal ultrasound (2DUS) is a routine and reliable diagnostic modality in the evaluation of uterine pathologies (2). However, even this advanced technology can only provide two dimensional views of three dimensional structures. The application of 3D ultrasound (3DUS) with increased spatial awareness in the last decade of the 20th century has enabled a detailed assessment of uterine morphology. 

## Methods

There are two methods for three dimensional volume acquisition: the freehand technique and automatic acquisition. With the freehand technique, images are obtained manually with the use of a two dimensional transducer. Decreased accuracy of measurements and less quality of images are two possible problems when comparing the freehand technique with the automated technique (2). 

We obtained the images in this pictorial review with the use of an ACCUVIX XQ, (Medison, Korea) and three-dimensional transvaginal 3D5-8EK probes. This system employs a newly introduced technique named 3DXI, which utilizes two modes: multi-slice view and oblique view.

1. The multi-slice view transforms three dimensional volume data obtained from a regular ultrasound scan into a series of sequential images captured at intervals of 0.5 mm to 5 mm segments. Users can instantly view, analyze and understand the more in depth data, and thereby gain greater confidence and accuracy.
2. The oblique view enables the operator to examine and view three dimensional volume data in various planes of view. The exact portion of three dimensional data that the operator wants to visualize can be selected, thus allowing for a more complete visual examination and better understanding of the correlation between organs and other areas within the region of interest.

### Structural pathology in the uterus

#### 1. Müllerian duct anomalies (MDAs)

MDAs are relatively common disorders associated with adverse reproductive outcomes. Critical analyses of studies suggest that the prevalence of uterine malformation in woman with repeated pregnancy loss is about 3% (3). Uterine malformation may result from arrested development of the Müllerian ducts, failure of fusion of the Müllerian duct or failure of resorption of the median septum (4, 5).

**Fig 1:**
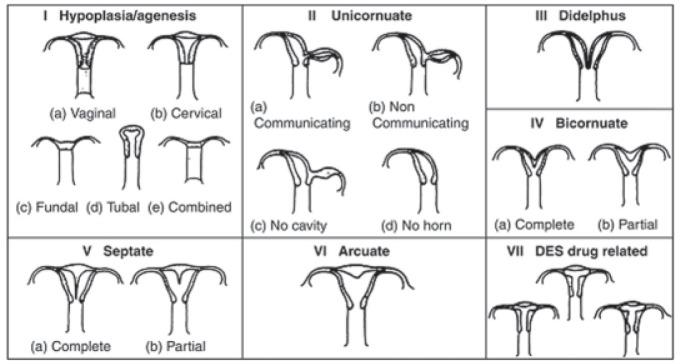
Classification system of Müllerian duct anomalies developed by the AFS.

Several classification systems describe MDAs. The most accepted system is the American Fertility Society (AFS) classification system (Fig 1).

According to AFS, class I consists of hypoplasia and agenesis. Class II represents unicornuate uterus. Categories III and IV are composed of uterus didelphys and bicornuate uteri. Categories V, VI and VII refer to septate uterus, arcuate uterus and diethylstilbestrol exposure, respectively (6, 7).

Various imaging modalities have been used to evaluate MDAs. Despite being invasive, hysteroscopy and laparoscopy are the conventional methods for assessing uterine morphology.

HSG is one of the first conventional diagnostic tools to provide valuable information about the uterine cavity and tubal patency; however, the diagnostic accuracy of hysterosalpingography (HSG) alone is only 55% in the differentiation of septate from bicornuate uteri (8). In addition, this procedure is associated with the following complications: radiation and iodinated contrast material exposures, as well as pelvic inflammatory disease (PID) and pain.

Technologic advances in imaging modalities have revolutionized the evaluation of MDAs by noninvasive tools such as 2DUS, 3DUS and magnetic resonance imaging (MRI).

Even though two dimensional sonography is routinely used because of its flexibility and moderate cost, it has some limitations. The sensitivity of 2DUS, particularly for the demonstration of fundal contour is relatively low compared with other methods (9). One of the most useful scan planes obtained on 3DUS is the coronal view of the uterus, which is usually not obtainable on 2DUS (10). Therefore, this view is a valuable problemsolving tool that assists in differentiating between various MDAs, including bicornuate, septate, unicornuate and didelphys uteri. Data acquisition time is short and images can be stored for later evaluation and analyzed as many times as necessary (9) (Figes2A-I).

Despite the high accuracy of MRI in appraising the cervix and vagina, some limitations may favor the use of 3DUS. MRI is highly operator and technique dependent, and costly. The most important advantages of real time 3DUS over MRI are lower cost, shorter examination time and wider availability (11). 3DUS has recently become the only mandatory step in classifying MDAs since there is a high degree of concordance between 3DUS and MRI in the diagnosis of uterine malformations.

3DUS images are easier to interpret in the luteal phase of the cycle due to increased thickness and echogenicity of the endometrium (9).

#### 2. Intrauterine lesions

Although fluid instillation into the endometrial cavity enhanced endometrial visualization during transvaginal ultrasound, intrauterine lesions (e.g., polyps, fibroids and synechiae) can be diagnosed sonographically in the initial investigation without the need for fluid instillation. Using 3DUS facilitates access to the most relevant plane, and ability to visualize both the endometrium and the myometrium facilitates correct diagnosis of uterine abnormalities.

3DUS affords a more comprehensive understanding of exact the numbers, sizes and locations of intrauterine lesions. Fibroids causing uterine distortion are the most common uterine abnormalities associated with subinfertility, implantation failure and miscarriage. One of the most useful views obtained on 3DUS are views that enable a clear estimation of the relationship between the endometrium and intramural fibroids, and consider whether they distort the uterine anatomy or not.

Intrauterine adhesions have a variable appearance, ranging from small echogenic areas of focal endometrial thickening to an obliterated endometrium as in Asherman’s syndrome.

**Fig 2:**
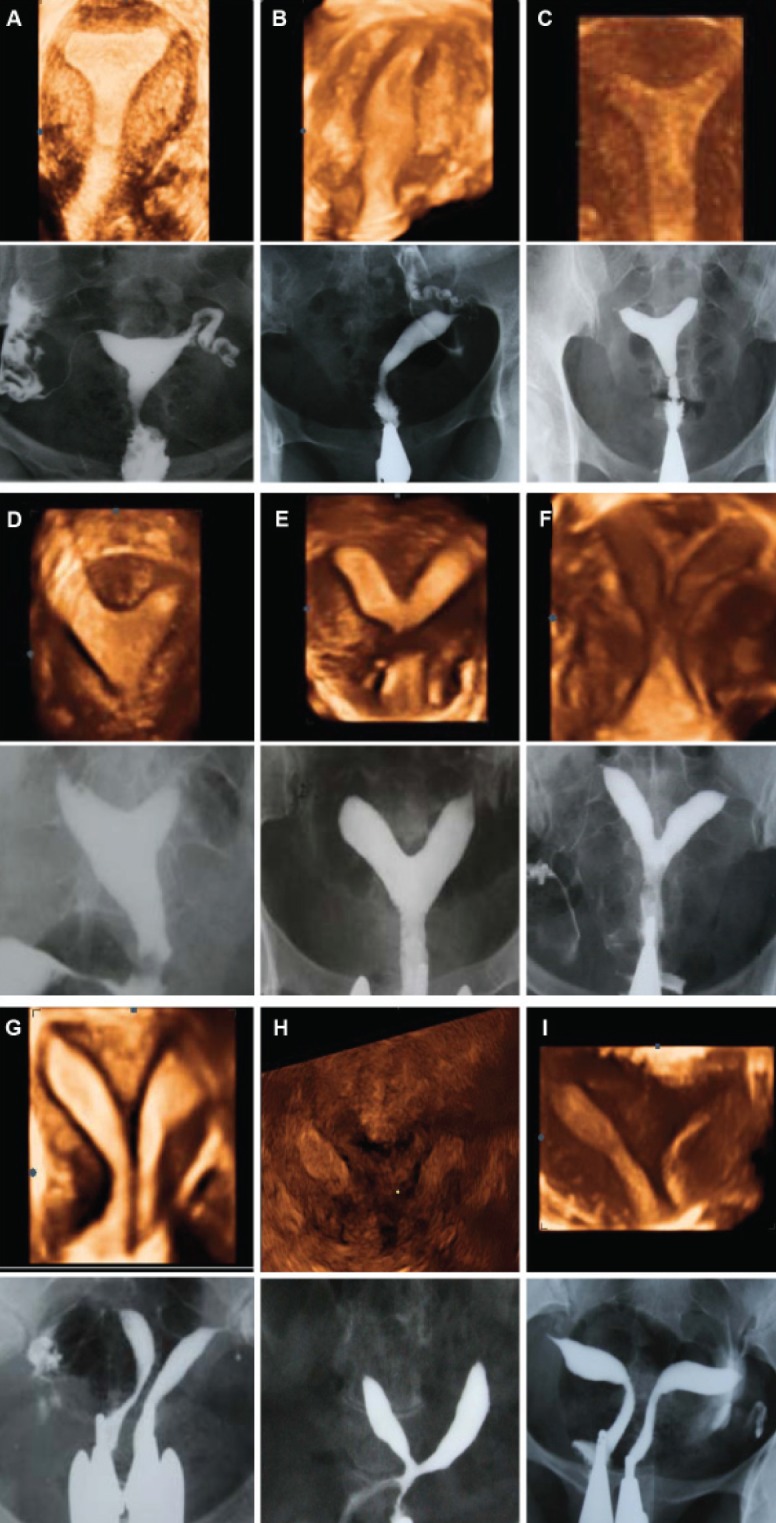
Comparison of three-dimensional ultrasound and HSG imaging in cases of uterine malformation using AFS: A. Normal uterus, B. Unicornuate uterus, C. Arcuate uterus, D-G. Different subtypes of septate uterus (partial to complete septum), H. Bicornuate uterus, I. Didelphys.

**Fig 3:**
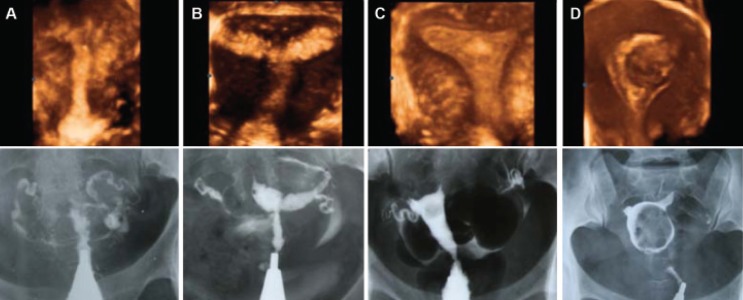
Comparison of three-dimensional ultrasound and HSG imaging in cases of intrauterine lesions: A. Obliteration of the uterine cavity due to severe synechiae, B. Moderately extensive synechiae involving ½ of the uterine cavity, C. Endometrial polyp in the fundal area, D. Marked distortion and deformity of the uterine cavity caused by an intramural myoma bulging to the cavity.

Endometrial polyps appear as persistent hyperechogenic areas with variable cystic spaces and distortion of endometrial contours. They are best visualized during the mid-cycle and not readily seen during the mid-luteal scan cycle when the endometrial layer is thick (12) (Fig 3A-D).

### Assessment of endometrial volume by three-dimensional ultrasound

3DUS, which has the ability to visualize surfaces three dimensionally, has recently become available for endometrial volume measurement. Ultrasonographic assessment of the endometrium is an important investigative tool in the assessment of endometrial receptivity. Endometrial volume is a useful criterion in predicting embryo implantation success and pregnancy rate in patients undergoing *in vitro* fertilization (IVF).

**Fig 4:**
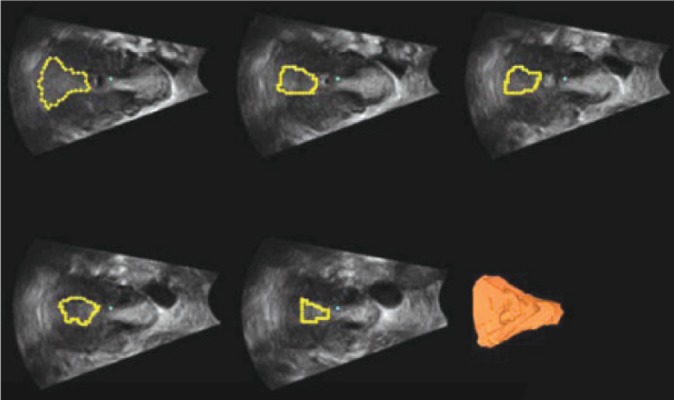
Three dimensional volume calculation of endometrium in multiplanar display.

Volume estimation of the endometrium is easily made because of good contrast between endometrial tissue and myometrium. 3DUS allows us to calculate the endometrial volume by manual delineation of the endometrium (13) (Fig 4).

## Conclusion

Endovaginal 3DUS is a non-invasive, outpatient diagnostic modality which enables a detailed assessment of uterine morphology and is superior to other techniques used for the same purpose. Because of the high level of agreement between 3DUS and hysterosalpingography, MRI, hysteroscopy and laparoscopy, therefore 3DUS has recently become the only mandatory step in the initial investigation of MDA and intrauterine lesions to assess whether hysteroscopic evaluation of the endometrial cavity is indicated.
